# Welding of S960QL High-Strength Steel by the Manual–Automated MAG Technique—A Study of Mechanical Properties, Residual Stresses and Fracture Mechanisms in the Heat-Affected Zone

**DOI:** 10.3390/ma17235792

**Published:** 2024-11-26

**Authors:** Tomasz Ślęzak

**Affiliations:** Institute of Robots & Machine Design, Faculty of Mechanical Engineering, Military University of Technology, ul. gen. S. Kaliskiego 2, 00-908 Warsaw, Poland; tomasz.slezak@wat.edu.pl

**Keywords:** high-strength steel, welded joints, residual stresses, impact toughness, microstructure, microhardness

## Abstract

This paper presents results of investigations of a V-type welded joint made of S960QL high-strength steel made using a mixed technique: the root was welded manually and the face automatically. Although high-strength steels have been available on the market for many years, they are still the subject of research due to their increasingly widespread usage. For this reason, detailed investigations of welded joints of S960QL steel were carried out in terms of microstructure, microhardness, impact toughness and residual stresses, in order to expand knowledge in this area. The obtained results made it possible to determine their changes in heat-affected zone (HAZ) as a function of the distance from the fusion line. One of the most important findings is that during the tensile tests, the rupture occurred in the sub-zone of HAZ, which is characterized by increased strength and low ductility. This was due to the fact that an unfavorable residual stress distribution occurred in this area, causing the highest initial local strain of the material. Furthermore, different fracture mechanisms, both ductile and brittle, as well as mixed, were observed and described in detail for each sub-zone of the HAZ and in the weld.

## 1. Introduction

The use of high-strength steels in various applications entails many benefits. Ultimately, these benefits can be described in three trends: reducing the costs and duration of the production process, improving the efficiency and performance parameters of machines and vehicles and reducing the negative impact on the environment. The first two advantages particularly affect the increase in the competitiveness of manufacturing and construction companies. This causes a continuous increase in interest in high-strength steels despite their higher price [1,2,3]. Moreover, the high-strength steel market is expected to grow steadily and its global value will reach USD 54 billion by 2027, exhibiting a CAGR (Compound Annual Growth Rate) of 7.4% during the forecast period [3]. The above-mentioned trends and forecasts clearly indicate that the interest in high-strength steels is constant and concerns various aspects. Numerous research centers are developing new types of steel with improved properties or intended for special applications, and the steels that have entered the market are subjected to continuous testing to learn about their performance characteristics. One of the areas of research into the performance properties of structural steels is the assessment of the influence of welding, which is currently the basic joining method used in steel structures.

The welding process, due to the input of heat causing the melting of the paternal material, generates numerous changes. The most important of them are local changes in the microstructure, mechanical characters and strength properties, the introduction of a geometric notch on the fusion line and the induction of a new state of residual stresses due to the solidification of the weld pool and phase transformations.

Microstructural and mechanical property tests form the basis for the analysis of the quality of welded joints. The production technology of high-strength steels makes them very sensitive to temperature effects, which is manifested by a weakening in the heat-affected zone (HAZ) due to the transformation of martensite into acicular ferrite with precipitating cementite [4,5,6]. Additionally, W. Guo et al. [7] showed that in the HAZ of laser-welded martensite–bainite S960 HSLA steel is a mixture of equiaxed martensite and auto-tempered martensite with a prior-austenite grain size in the coarse-grained (CGHAZ) and fine-grained sub-zones (FGHAZ). At a greater distance from the fusion line (FL), the structure consists of an overtempered base material microstructure and regions with high carbon martensite and auto-tempered martensite. W. Li et al. [8] presented the results of the comparative metallographic analysis, microhardness testing and tensile testing of HC420LA steel welded with a laser beam and MAG, in order to optimize laser welding parameters. Microstructure tests are almost always supplemented by microhardness measurements and tensile tests [8,9,10,11]. These three types of tests provide the basis for assessing whether a welded joint has been made properly and should always be performed.

The area of mechanical property testing is complemented by the impact test. Impact toughness allows researchers to determine the ductility of a material, which is very important for predicting the type of cracking and the rate of crack growth at different temperatures. Steels with too low impact toughness are not permitted for use in welded structures. The authors of [12] presented the impact properties of S700MC steel and its welded joint at different impact energies. However, it was not indicated where exactly the impact test was performed in the welded joint. In [13], the properties of welded joints subjected to dynamic interactions were investigated. An important issue is the assessment of impact toughness at different temperatures in simulated welding cycles or at different distances from the fusion line [14,15,16,17]. This allows for the assessment of the impact properties of different HAZs, especially in CGHAZ.

A significant problem in welded joints are the residual stresses generated in the weldment and its vicinity during the welding process. Incorrectly selected welding parameters can lead to hot cracking [18,19] or hydrogen-induced cold cracking [20,21] or cause an unfavorable stress distribution resulting in rapid failure during operation [22,23,24] or during repair processes [25]. For this reason, the residual stresses in welded joints of high-strength steels are constantly the subject of research.

With reference to the above-mentioned research areas, it was recognized that it is still desirable in the research environment to deepen the knowledge of the basic properties of welded joints of HSS steel. This applies especially to the examination areas of microstructure, microhardness and impact toughness, as verification tests of the adopted welding conditions for a specific steel grade. Moreover, the research area was extended to include a detailed analysis of the residual stress state in the HAZ.

## 2. Material and Methodology

### 2.1. Tested Material

The study was conducted on a fine-grained high-strength structural steel S960QL purchased in the form of a sheet with a thickness of 6 mm. This steel is manufactured through a process of rolling and heat treatment, which results in a fine-grained martensite–bainite structure with the equivalent diameter of grains being from 10 to 25 μm [26]. Both upper and lower bainite were found. This structure is relatively stable in thermal processes like welding and improves the strength properties, which is a crucial factor.

The chemical composition of this steel is shown in Table 1, which presents the values from the manufacturer’s certificate and from the author’s own measurement made using a JSM-6610 scanning electron microscope (Jeol Ltd., Tokyo, Japan) equipped with an X-Max 50 EDS spectrometer (Oxford Instruments NanoAnalysis, HighWycombe, UK).

The strength properties were determined during a tensile test according to the standard ISO 6892-1:2016 [27] and the values included in the certificate were confirmed. Moreover, additional properties were specified and all data are placed in Table 2, where E—Young modulus; σ_PL_—proportionality limit; σ_Y_—yield strength; σ_U_—ultimate tensile strength; σ_T_—fracture strength; L—elongation at fracture; Z—necking ratio. The tests were conducted using an Instron 8802 hydraulic pulsator (Instron, Norwood, MA, USA) equipped with an Instron 2630-112 extensometer with a gauge length of 50 mm. The samples were cut in the direction of rolling.

**Figure 1:**
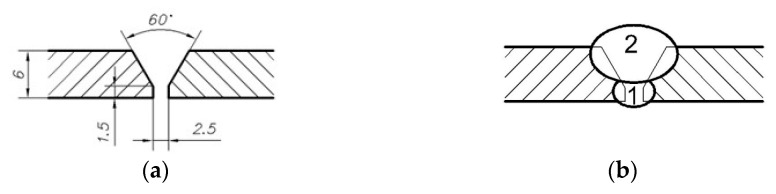
Dimensions of joint before welding (**a**) and the sequence of passes (**b**).

The single-V butt joints were made using the MAG welding process using the following consumables: M21 shielding gas containing 82% CO_2_ with 18% Ar (M21–ArC–18), and welding wire UNION X 96 (voestalpine Böhler Welding Group GmbH, Düsseldorf, Germany) [28] (G 89.5 Mn4Ni2.5CrMo) with a diameter of 1.2 mm. A geometry of joints before welding and a sequence of welding layers are presented in Figure 1. Additionally, the parameters of welding are shown in Table 3.

The first pass was made manually, contrary to the second, which was made in a robotized manner, but both passes were executed in a flat position. The parameters of welding were determined in order to not exceed a value of heat input of 1 kJ/mm.

All joints were radiologically controlled using the Seifert Eresco 65 MF3 X-ray apparatus (Waygate Technologies, Ahrensburg, Germany). The quality of the welds was assessed according to the ISO 5817 [29] standard at the “B” level, which corresponds to the highest requirements for welded joints.

A sample for microstructure investigation was cut out of the welded joint perpendicularly to the weld axis. Next, it was ground to 2000 grade, polished and etched with 5% nital solution. Finally, the structure was observed using a OLYMPUS LEXT OLS 4100 laser scanning digital microscope (Olympus, Hamburg, Germany).

### 2.2. Mechanical Properties

Tensile tests were conducted using an Instron 8802MTL universal testing machine with the WaveMatrix computer software (1.8.383.0, Instron, Norwood, MA, USA), and three tests were performed. A 2620-604 extensometer (Instron, Norwood, MA, USA) with a measurement base of 50 mm was used to measure the strains. Samples were prepared in accordance with the ASTM E8/E8M-13a standard [30] but it should be noted that the face and root reinforcements were not removed.

Microhardness was measured by the Vickers method using a semi-automatic micro-hardness tester (Shimadzu, Kyoto, Japan) in accordance with standards [31,32,33]. The HV0.1 tests were carried out on the cross-sectional surfaces of the weldments in two rows of indentations which were parallel to the surfaces and spaced approx. 0.5 mm from them. The surface was prepared by grinding and polishing.

**Figure 2:**
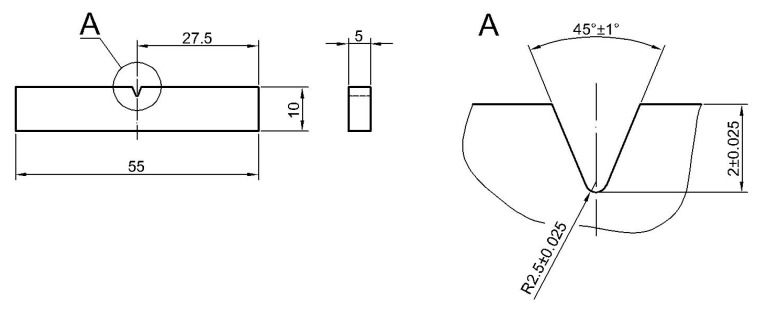
Dimensions of the samples used in the impact test.

The impact test was carried out at room temperature by the Charpy method, in accordance with the standard [34,35], using an Wolpert PW30 (Instron, Norwood, MA, USA) impact testing machine. In the test, samples made of paternal material were used after welding, in which notches were located in different zones of the welded joint. Taking into account the type and thickness of material, the samples machined with a “V” type notch and a reduced thickness. A geometry is presented in Figure 2.

**Figure 3:**
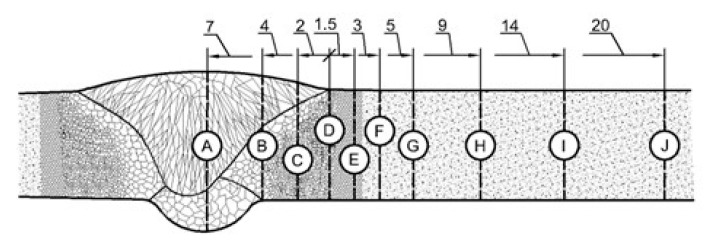
Location of notches and distances from the fusion line in the samples used during the impact tests; unit: [mm].

The placement of the notches vs. the zones of weldment scheme is shown in Figure 3. The notches were located in the axis of the weld (A); at the fusion lines on a root side (B) and on a face side (D); halfway between B and D (C); and at six different distances from the fusion line measuring on the face side FFL (E–J).

Before testing, each sample was inspected on a stand equipped with a micrometric dial indicator, during which its transverse dimensions and the depth of the notch were carefully verified.

### 2.3. Residual Stresses

**Figure 4:**
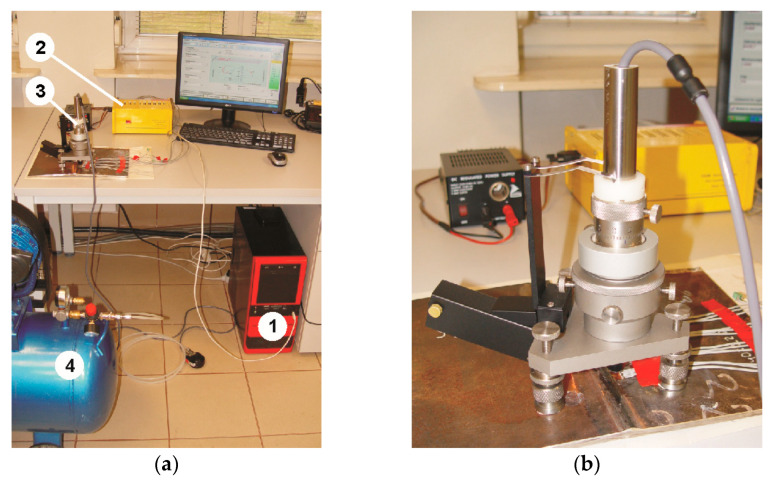
Measuring station for determining residual stresses (**a**) and RS-200 device prepared for drilling (**b**): 1—central unit; 2—ESAM Traveler Plus strain gauge bridge; 3—RS-200 device; 4—supplying compressor.

The residual stresses were determined using the hole-drilling method. This method allows researchers to determine the change in the state of residual stresses with the increase in depth and to assess the nature of these changes. The measurements are aimed at determining the state of stress and their change depending on the distance from the fusion line. The methodology of determining residual stresses by the hole-drilling method has been standardized and described in ASTM E 837 [36]. The precise execution of the hole during the measurement was ensured by the use of a dedicated RS-200 Milling Guide device (Vishay Precision Group, Malvern, PA, USA). The practice of performing measurements using the above device and the methodology for determining stresses were described in the Tech Note TN-503 [37]. The holes were drilled using carbide cutters with a diameter of 1.60 mm. The obtained voltage output signal was transmitted from the rosettes to the channels of the ESAM Traveler Plus type 1032-S strain gauge bridge (ESA Messtechnik GmbH, München, Germany). The values of the principal stresses and their angular orientation were determined using the H-Drill software (3.21, Gary S. Schajer, Vancouver, BC, Canada). The residual stress measurement station is shown in Figure 4.

The holes were drilled to a depth of 2 mm, recording the output voltage changes in 0.1 mm increments. The values of the recorded voltage output signal were converted into strain using the formula (1):

ε = 4 · U_OUT_ · (U_0_ · N · K · A)^−1^ (1)

where:

U_OUT_—output voltage [V];
U_0_—input voltage [V];
N—coefficient depending on the bridge type, N = 1;
K—strain gauge constant;
A—amplification coefficient.

In order to determine the principal stresses and their angular orientation, the following formulas were used [37]:

(2) $$\sigma_{max}=\frac{\varepsilon_{1}+\varepsilon_{2}}{4\cdot A}-\frac{1}{4\cdot B}\sqrt{{\left(\varepsilon_{3}-\varepsilon_{1}\right)}^{2}+{\left(\varepsilon_{3}+\varepsilon_{1}-2\varepsilon_{2}\right)}^{2}\;}\left[MPa\right]$$

(3) $$\sigma_{min}=\frac{\varepsilon_{1}+\varepsilon_{2}}{4\cdot A}+\frac{1}{4\cdot B}\sqrt{{\left(\varepsilon_{3}-\varepsilon_{1}\right)}^{2}+{\left(\varepsilon_{3}+\varepsilon_{1}-2\varepsilon_{2}\right)}^{2}\;}\left[MPa\right]$$

(4) $$\alpha =\frac{1}{2}arctg\frac{\varepsilon_{1}-2\varepsilon_{2}+\varepsilon_{2}}{\varepsilon_{2}-\varepsilon_{1}}\left[MPa\right]$$

where:

σ_max_, σ_min_—principal stresses;
ε_1_, ε_2_, ε_3_—strains determined in individual strain gauges of the rosette;
A, B—coefficients depending on the material properties and geometry of the rosette and hole;
α—angle between no. 1 strain gauge and the nearest principal stress.

The values of the principal stresses and their angular orientation were determined using the H-Drill software.

## 3. Results and Discussion

### 3.1. Microstructure

**Figure 5:**
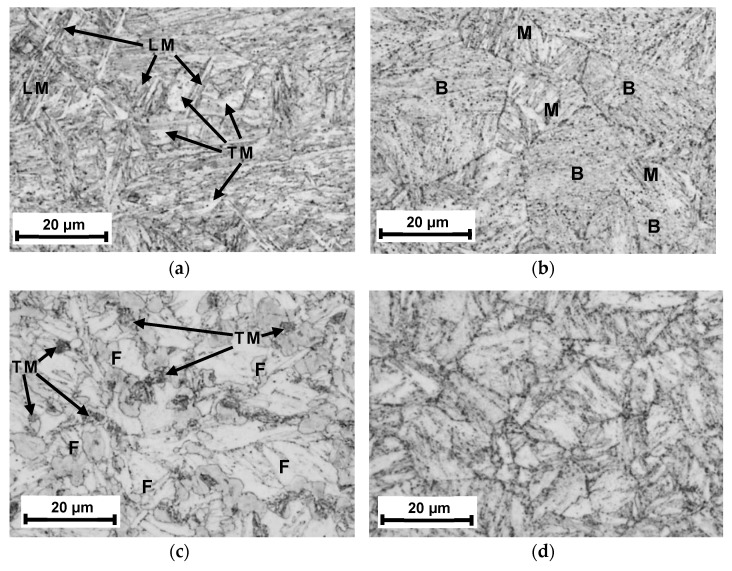
Macrostructure of the V-type welded joint of S960QL steel—the sub-zones of the HAZ: CGHAZ (**a**), FGHAZ (**b**), ICHAZ (**c**) and SCHAZ (**d**).

The results of the microstructure investigation are presented in Figure 5. The specific sub-zones of the HAZ are presented in Figure 5a–d, namely, coarse-grained CGHAZ (5a), fine-grained FGHAZ (5b), intercritical ICHAZ (5c) and sub-critical SCHAZ (5d).

The quality of the joint was high: it had completely melted through the welded material and no macroscopic defects were observed, such as cracks, porosity, overlap or inclusions. The microstructure of the CGHAZ was a mixture of lath martensite (LM) and tempered martensite (TM), both with larger grain sizes [38]. The microstructure of the FGHAZ (Figure 5b) mainly consisted of fine-grained tempered martensite–bainite (B) mixed with some martensite, both lath and tempered (M) [38]. In the next zone, namely, the ICHAZ, incomplete normalization was taking place. Due to the specific thermal cycle, the partially transformed austenite transferred into tempered martensite (TM) after cooling, while the untransformed ferrite (F) only underwent heating and growth [39]. The SCHAZ was characterized by a microstructure similar to that of the paternal material. In all photos of the structure, one can see precipitates in the form of carbides (black points).

Such a diverse microstructure in the HAZ resulting from different thermal cycles affects mechanical properties. Lath martensite as a result of hardening has high hardness and low susceptibility to deformation, which affects its brittleness. In turn, tempered martensite/bainite, due to the occurrence of diffusion processes, is characterized by very good strength properties, lower hardness, excellent plasticity, impact strength and load-bearing capacity. Austenite occurring in the zone of incomplete normalization is characterized by low hardness, and the inconsistency of the crystal lattice (body-centered in austenite vs. face-centered) causes a decrease in mechanical strength. Regardless of the detected structural phase, grain growth always results in a decrease in strength properties, including impact strength. For this reason, in the subsequent part of the study, microhardness and impact strength measurements were carried out in individual HAZ subzones to observe the magnitude of their changes and to compare the nature of the changes with the general features of the structural phases described above. The study was supplemented by measurements of residual stresses, which, in the zone of incomplete normalization, should be tensile, due to the difference in the crystallographic structure.

### 3.2. Mechanical Properties

The results of the conducted tensile tests of V-type welded joints of S960QL steel allowed the author to determine the values of the basic strength parameters of this steel in the as-welded condition. They are as follows: yield strength σ_Y_ = (939 ± 7) MPa; ultimate strength σ_U_ = (1079 ± 7) MPa; and elongation at fracture EL = (9 ± 0.6) %. For each tested sample, the necking occurred in the HAZ, while the fracture place was located approximately 3 mm from the FFL. The obtained results indicate that the strength of this steel is very good and the yield strength σ_Y_ reaches over 95% of the nominal value of the paternal material (according to the standardized steel grade designation “960”).

**Figure 6:**
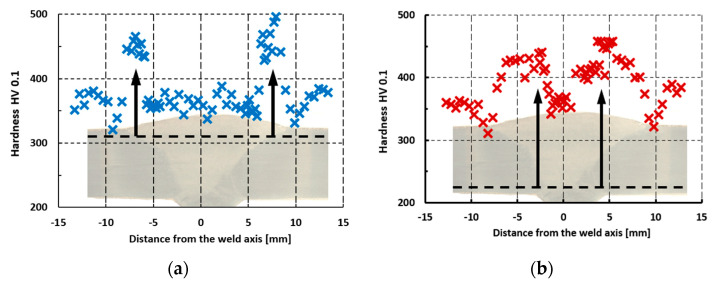
Microhardness changes in the welded joint of S960QL steel with a “V” weld on the face side (**a**) and on the root side (**b**).

The results of the microhardness measurements are presented in Figure 6, where the thumbnails of the weld view are placed too. Black dashed lines indicate the rows of indentations and the arrows point out the results in the vicinity of the fusion lines.

Based on the measurement results presented in Figure 6, it can be seen that the highest microhardness in the joint zone is located in the immediate vicinity of the fusion line, where the hardness reaches almost 500 HV0.1 (Figure 6a). Tempering zones were observed in the HAZ, characterized by a decrease in microhardness to a value of 310–330 HV0.1, where incomplete normalization occurred (ICHAZ). The beneficial effect of the second welding pass is noticeable, smoothing the hardness gradient in the first pass (Figure 6b). This supports the tempering passes presented in the literature on the multi-pass welding of structural steels [11,15,16]. It is worth noting that in the joint zone located between the fusion lines and in the area outside the HAZ, the microhardness is in the range of 340–380 HV0.1.

The Charpy impact test results are presented in Table 4. Two measurements were taken on the fusion line, both from the root side and from the face side. The impact strength of the paternal material (PM) was determined based on the results of three tests.

**Figure 7:**
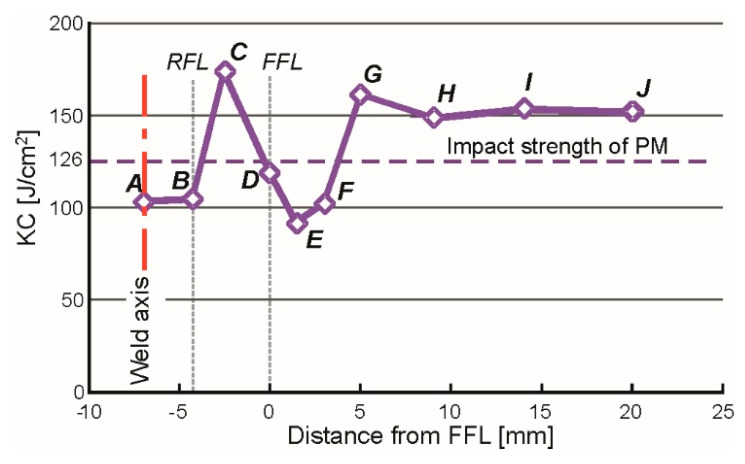
Changes in the impact toughness (KC) in the weld and HAZ of the V-type joint of the S960QL steel.

The obtained results were used to develop a graph of impact strength changes (Figure 7). The graph shows the locations of the fusion line on the root side (RFL) and on the face side (FFL) with vertical dashed lines. The impact strength value in the paternal material was 126 J/cm^2^.

A significant reduction in impact toughness can be observed within 3 mm of the FFL line (points D–F), which is related to the ICHAZ where the incomplete normalization occurred. Next, there is a significant increase in KC in the SCHAZ (point G), followed by a slight decrease and stabilization after reaching a value of about 150 J/cm^2^. This value is higher than the value determined for the paternal material, which is probably due to the slight heating-up of the original martensitic–bainitic structure of this steel.

**Figure 8:**
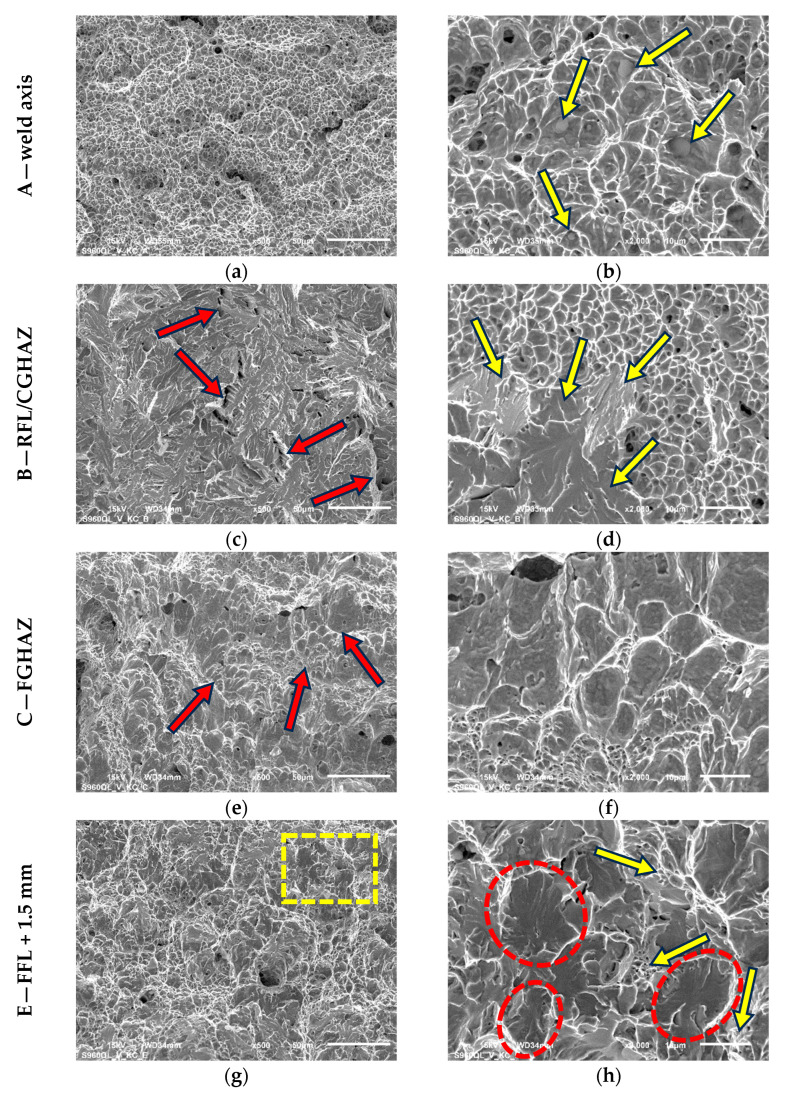

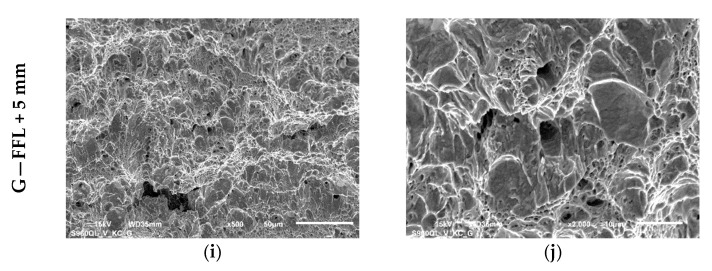
The fracture surfaces of selected samples after impact tests shown in two magnifications. Photos taken in the central part of the fracture surface of weld axis (**a**,**b**) and different subzones of HAZ: coarse-grained (**c**,**d**), fine-grained (**e**,**f**), intercritical (**g**,**h**), subcritical (**i**,**j**).

The fractures of the samples obtained after the impact tests were subjected to fractographic examinations in order to explain the destruction mechanisms in the melted area of the weld and individual sub-zones of the HAZ. Observations were made using a JSM-6610 scanning electron microscope (Jeol Ltd., Tokyo, Japan) and were carried out for samples A, B, C, E and G. The results are shown in Figure 8.

As a result of the impact test of the weld, an irregular fracture was obtained, characterized by densely distributed small dimples (Figure 8a). This means that the decohesion of the material occurred simultaneously over a large area and plastic deformation was very limited. Numerous spheroidal inclusions are responsible for this nature of fracture (Figure 8b). They were observed only at the weld fracture, which indicates that they are of welding origin. This was confirmed by further study revealing a significant presence of oxygen. The EDS analysis of these inclusions showed that they contain Mn, O, Ti, S and Al. Based on the results presented in [40,41,42], it can be concluded that there can be numerous types of particles, i.e., (Mn, Al)-oxides, Mn-silicate or the phase Mn(Al,Ti)_2_O_4_ commonly called galaxite. They constitute both the origins of grain nucleation and welding inclusions. The places where these inclusions occurred became the initiators of the development of microcracks, which after a slight growth merged into a macroscopic crack. Although the surface structure is characteristic of ductile fracture, the indicated features contributed to the reduction in impact strength. A different fracture mechanism was revealed in the CGHAZ, though this sub-zone is characterized by the same impact strength as the weld. Large fields of a brittle transcrystalline fracture were recorded as characteristic of this zone, with local intercrystalline cracks (Figure 8c). Figure 8d represents the transition zone between transcrystalline and ductile fracture with small dimples, where the brittle fracture planes were very well exposed (arrows). A completely different crack development mechanism was observed in FGHAZ, where the fracture surface exhibited the nature of ductile cracking (Figure 8e,f). In this case, there are slightly outlined fractographic mesostructure lines, indicating a temporary cessation of cracking (red arrows). This proves a greater possibility of energy dissipation in the material and that the accumulation of impact energy occurred in stages. The ductile nature of the fracture is very well visible in Figure 8f in the form of elongated dimples. As a consequence, the highest impact strength occurs in this location. A completely different mechanism of cracking was defined in the case of the impact test performed at the ICHAZ (Figure 8g,h), which is characterized by the lowest impact toughness. The macroscopic view shows features of ductile fracture, with dimples of various sizes formed. A more detailed analysis of the fracture surface (the area indicated in Figure 8g) revealed a complex ductile–brittle fracture nature. Ductile cracking developed only in the matrix of the grains, which was manifested by characteristic areas with small dimples (yellow arrows). However, transcrystalline cracks were revealed in the grains (examples—red ovals in Figure 8h). Such a mechanism is characteristic of the incomplete normalization zone (ICHAZ). In the SCHAZ (Figure 8i,j), the fracture mode is very similar to that described for the FGHAZ.

### 3.3. Residual Stresses

**Figure 9:**
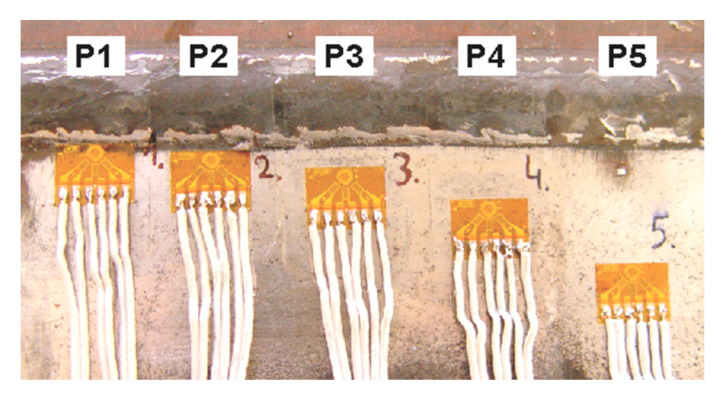
Welded joint of S960QL steel with the rosettes for measuring residual stresses in the HAZ located on the face side.

The measurement of residual stresses in the HAZ of the “V” type weld was carried out at five points using CEA-06-062UM-120 rosettes. The view of the rosettes and their arrangement are shown in Figure 9. The first measuring point (P1) was located directly on the fusion line, while the other ones (P2–P5) were located at distances of 2, 5, 10 and 20 mm from the fusion line.

Based on the recorded measurement results, the values of the principal stresses were calculated. Due to the fact that in some cases the stresses were not uniform, i.e., their magnitude and direction changed with depth, this article presents results corresponding to a layer of 0.1 mm depth (Table 5). This approach seems justified, since surface defects are the most important for the performance properties of joints in terms of strength (e.g., the effects of cyclic loading). Residual stresses may additionally intensify the impact of geometric or structural notches.

**Figure 10:**
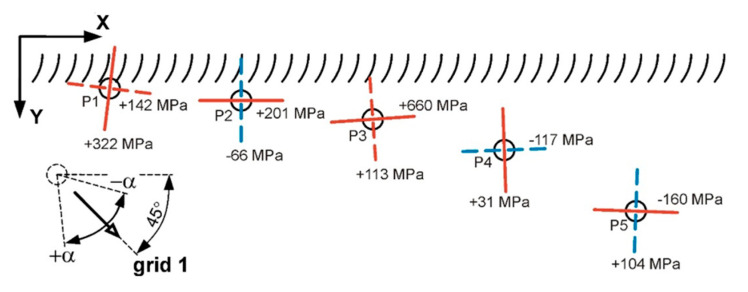
The graphical representation of the residual stresses measurement results in the vicinity of the weld, where a continuous line—σ_max_; a dashed line—σ_min_; blue color—compressive stresses; red color—tensile stresses.

In the analysis of the results, the best fit was obtained in the case of using the power series method. With this approach, the error in fitting the recorded strain changes to the theoretical values was the lowest. This table also provides the 90% probability bound values for each calculated quantity (P_90%_), which also takes into account the absolute hole diameter measurement error of 0.04 mm and the Young modulus determination error of 1%. The graphical presentation of the residual stress measurement results is given in Figure 10. The directions of the principal stresses and their values, as well as the orientation of the axis of the strain gauge no. 1 of the measuring rosettes (grid 1), are plotted there.

**Figure 11:**
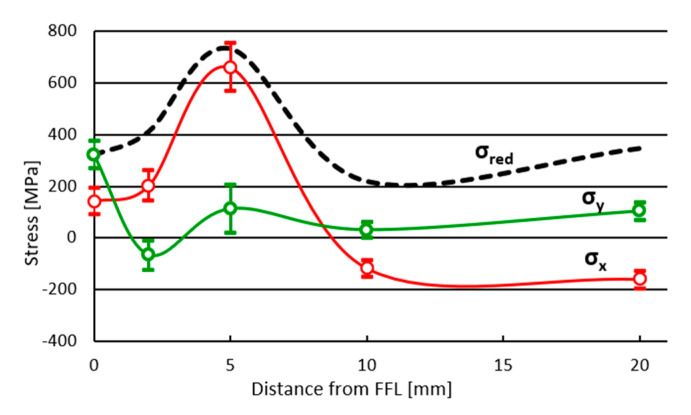
The course of stress changes in the heat-affected zone: longitudinal σ_x_, transverse σ_y_ and reduced σ_red_.

The obtained measurement results allowed the author to draw a graph of changes in welding stresses, namely, longitudinal σ_x_, transverse σ_y_ and reduced σ_red_, as a function of distance from the fusion line, presented in Figure 11. The reduced stresses σ_red_ were determined based on the HMH hypothesis using the formula (5):

(5) $$\sigma_{red}=\frac{1}{\sqrt{2}}\sqrt{{\left(\sigma_{11}-\sigma_{22}\right)}^{2}+{\left(\sigma_{22}-\sigma_{33}\right)}^{2}+{\left(\sigma_{11}-\sigma_{33}\right)}^{2}+6\left(\tau_{12}^{2}+\tau_{23}^{2}+\tau_{13}^{2}\right)}$$

In the analyzed case, the above formula takes a simplified form (6):

(6) $$\sigma_{red}=\sqrt{\sigma_{x}^{2}+\sigma_{y}^{2}-\sigma_{x}\sigma_{y}+3\tau_{xy}^{2}}$$

The determined nature of changes in residual stresses in the HAZ is similar to that presented in the literature [43,44,45]. The directions of the principal stresses of the stress tensor are very similar to the directions of the welding stresses σ_x_ and σ_y_, which causes them to reflect the values of longitudinal and transverse stresses. The stress σ_y_, apart from a local change in sign at a distance of about 2 mm from the fusion line, is characterized by stability and reaches a positive value in the HAZ. Such a distribution is caused by the transverse shrinkage of the weld, causing the tensile stress of the heated material zone. The stress value σ_x_ increases from the fusion line, where it is 142 MPa, to a maximum value of 660 MPa at a distance of about 5 mm, after which it decreases rapidly, transforming into a compressive stress of −117 MPa at a distance above 10 mm from the fusion line. This phenomenon is caused by the longitudinal shrinkage of the solidifying and then cooling weld, which results in compressive stresses outside the heating zone. It should be emphasized that the welding stress values change depending on the longitudinal location where the measurement is taken [46,47]. To minimize this effect, measurements were taken in the middle of the weld seam, at a distance of approx. 80 mm. It is worth noting that the maximum material effort caused by the residual stress state is located at a distance of about 5 mm from the fusion line and is represented by the local maximum σ_y_.

Analyzing the influence of the observed and identified structure in HAZ on mechanical properties, characteristic relationships can be described. In the CGHAZ, lath martensite occurred (Figure 5a), causing a significant increase in hardness (Figure 6), and together with grain growth, it affected the reduction in impact strength (Figure 7), caused by the brittleness of the lath structure. In the FGHAZ, however, the material was not overheated, but a normalization process occurred. As a result, a bainitic structure was formed in the tested steel (Figure 5b). This type of structure is characterized by very good strength properties, mainly very high impact strength, higher than that of the paternal material, and high hardness. This is due to the characteristic structure: the lamellar ferritic structure is reinforced with fine-dispersed carbide (cementite) precipitations.

The presented research results indicate that a severe decrease in hardness and impact strength occurred in the ICHAZ (Figure 6 and Figure 7), which is caused by the incomplete normalization of the steel. In this zone, a particularly unfavorable phase arrangement was observed. In the low-strength ferritic matrix, a few fine-tempered martensite grains were observed (Figure 5c) and relatively large carbides, which did not improve strength but caused the matrix to be depleted of carbon. Despite the lowest strength, which can be concluded from the hardness measurements [48], the fracture in the tensile test occurred at a distance of approx. 3 mm from the FFL, i.e., in the transition zone between the ICHAZ and the SCHAZ, where the hardness is significantly higher, but impact strength is still low; however, it is increasing. This is caused by local material strain. In this zone, the residual stresses are growing rapidly, especially longitudinal σ_x_ (Figure 11). Loading the sample with an axial force causes the summation of external stresses with transverse residual stresses. As a result, the limit state of material effort in a triaxial state of stress was reached the fastest in this zone and a rupture occurred. The described phenomenon can be avoided by using the post-process treatment of welded elements, e.g., by annealing or ultrasonic vibration. Even heating to a temperature of 400 °C can significantly reduce residual stresses [49]. However, it should be remembered that the careless heating of high-strength steels obtained as a result of thermomechanical rolling processes can result in a decrease in strength properties.

## 4. Summary and Conclusions

In this paper, a V-type welded joint made of HSS S960QL steel was subjected to a thorough analysis. It was made using a manual–automated MAG technique, i.e., the root was welded manually and the face in a robotic manner. The structural changes in individual zones of the welded joint and the influence of these changes on impact strength and microhardness are described in detail. After the impact test, the samples were subjected to fractographic analyses, which allowed for the description of the fracture mechanisms in each of the HAZ sub-zones. This work was enriched with the results of residual stress measurements, which allowed the author to explain the cause of fracture of tensile samples in the transition zone between the ISHAZ and the SCHAZ. Based on the obtained results, the following conclusions can be drawn:

(i) Four sub-zones were determined in the HAZ, namely, coarse-grained (CGHAZ), fine-grained (FGHAZ), intercritical (ICHAZ) and sub-critical (SCHAZ), among which the ICHAZ showed the worst strength properties because of incomplete normalization.
(ii) The highest ductility parameter KC was recorded in the FGHAZ with a value of 174 J/cm^2^, followed by the SCHAZ with a value of 161 J/cm^2^. The zone with the lowest impact strength of 92 J/cm^2^ (ICHAZ) also had the lowest microhardness value of 310–330 HV0.1.
(iii) The residual stresses were obtained using the hole-drilling method and had a characteristic distribution in the heat-affected zone. The highest value of residual stress was recorded in the SCHAZ (approx. 5 mm from the FFL), both for the longitudinal and reduced stresses, exceeding, respectively, 600 MPa and 700 MPa.
(iv) The high quality of the welded joint obtained was confirmed in the tensile test. Rupture occurred in the transition zone between the ICHAZ and the SCHAZ, which is characterized by increased strength and lower ductility, but in this zone, rapidly growing residual stresses occurred, causing the highest initial local strain of the material.

## Figures and Tables

**Table 1:** Chemical composition of S960QL steel.

	Elements [wt %]									
	Al	Si	V	Cr	Mn	Ni	Cu	Mo	C	Fe
By certificate	0.08	0.28	0.03	0.22	1.13	0.08	0.18	0.67	0.18	balance
By measurement	0.11	0.36	0.03	0.23	1.19	0.06	0.19	0.66	*	balance

* Value from certificate.

**Table 2:** Strength properties of S960QL steel.

	Strength Properties						
	E	σ_PL_	σ_Y_	σ_U_	σ_T_	L	Z
	[MPa]	[MPa]	[MPa]	[MPa]	[MPa]	[%]	[%]
By certificate	---	---	997	1069	---	13	---
By measurement	2.2 × 10^5^	917	974	1070	658	14.2	45.6

**Table 3:** Welding parameters of the single-V butt joints.

Number of Passes	Current	Arc Voltage	Wending Speed	Wire Feed Rate	Shielding Gas Flow	Heat Input
	[A]	[V]	[mm/min]	[m/min]	[l/min]	[kJ/mm]
1	150	19.5	210	5.5	14	0.67
2	260	27.0	450	7.5	14	0.75

**Table 4:** Results of Charpy V-notch test obtained in the tests of the V-joint of S960QL steel.

Position	A	B		C	D		E	F
Cross-section [mm^2^]	39.21	39.77	39.66	39.11	39.58	39.48	39.73	39.60
Absorbed energy [J]	40.5	40.0	43.5	68.0	50.0	44.0	36.5	40.5
Impact strength KC	103	101	110	174	126	111	92	102
**Position**	**G**	**H**	**I**	**J**	**PM**			
Cross-section [mm^2^]	39.06	39.66	40.02	39.42	39.70	38.67	38.94	
Absorbed energy [J]	63.0	59.0	61.5	60.0	49.5	49.5	49.0	
Impact strength KC	161	149	154	152	125	128	126	

**Table 5:** Results of residual stress measurement.

Measuring Points	Principal Stresses				Shear Stresses		Angle	
	σ_max_ [MPa]	P_90%_	σ_min_ [MPa]	P_90%_	τ_max_ [MPa]	P_90%_	α [ ° ]	P_90%_
P1	322	377 272	142	194 91	90	143 39	52	55 50
P2	201	261 146	−66	−9 −124	134	193 78	−45	−45 −45
P3	660	757 571	113	206 21	274	368 182	−48	−49 −47
P4	31	63 1	−117	−87 −150	74	106 44	48	49 48
P5	104	138 71	−160	−128 −195	132	167 99	46	46 46

## Data Availability

The original contributions presented in the study are included in the article material, and further inquiries can be directed to the corresponding author.
